# Supplementary material for: Evaluation of an mHealth-enabled hierarchical diabetes management intervention in primary care in China (ROADMAP): A cluster randomized trial
Source: PLoS Med. 2021 Sep 21;18(9):e1003754. doi: 10.1371/journal.pmed.1003754 (PMC8454951; doi:10.1371/journal.pmed.1003754)
Supplement: S1 Fig — EOS, end of study; HbA1c, glycated hemoglobin. (PDF) [file pmed.1003754.s002.pdf]

Subgroups	Control (n, %)	Intervention (n, %)	Relative Risk (95%CI)	P for Heterogeneity
**Age group**				
<60 years	777 (34.5%)	1966 (41.6%)	1.260 (1.151, 1.371)	0.067
≥60 years	1394 (39.5%)	3154 (45.0%)	1.147 (1.063, 1.232)	
**Sex**				
Male	783 (34.1%)	1956 (40.7%)	1.200 (1.093, 1.310)	0.986
Female	1388 (39.8%)	3164 (45.7%)	1.181 (1.095, 1.267)	
**Region**				
Developed	1045 (40.5%)	2359 (44.6%)	1.131 (1.022, 1.242)	0.298
Less Developed	1126 (35.2%)	2761 (42.8%)	1.238 (1.122, 1.357)	
**Residence locality**				
Urban	1212 (41.4%)	2690 (45.9%)	1.120 (1.023, 1.217)	0.135
Rural	959 (33.6%)	2430 (41.4%)	1.275 (1.143, 1.410)	
**Duration of diabetes**				
<6 years	1276 (45.9%)	2860 (51.0%)	1.122(1.044, 1.199)	0.05
≥6 years	895 (29.8%)	2259 (36.9%)	1.275 (1.163, 1.390)	
**Diabetic complication**				
Yes	708 (38.3%)	1623 (43.3%)	1.144 (1.043, 1.248)	0.261
No	1463 (37.2%)	3451 (43.8%)	1.207 (1.117, 1.297)	
**HbA1c level**				
<8%	1844 (53.0%)	4170 (59.6%)	1.127 (1.067, 1.186)	0.066
≥8%	327 (14.2%)	950 (20.0%)	1.464 (1.249, 1.706)	
**Total**	2171 (37.5%)	5120 (43.6%)	1.186 (1.105, 1.267)	

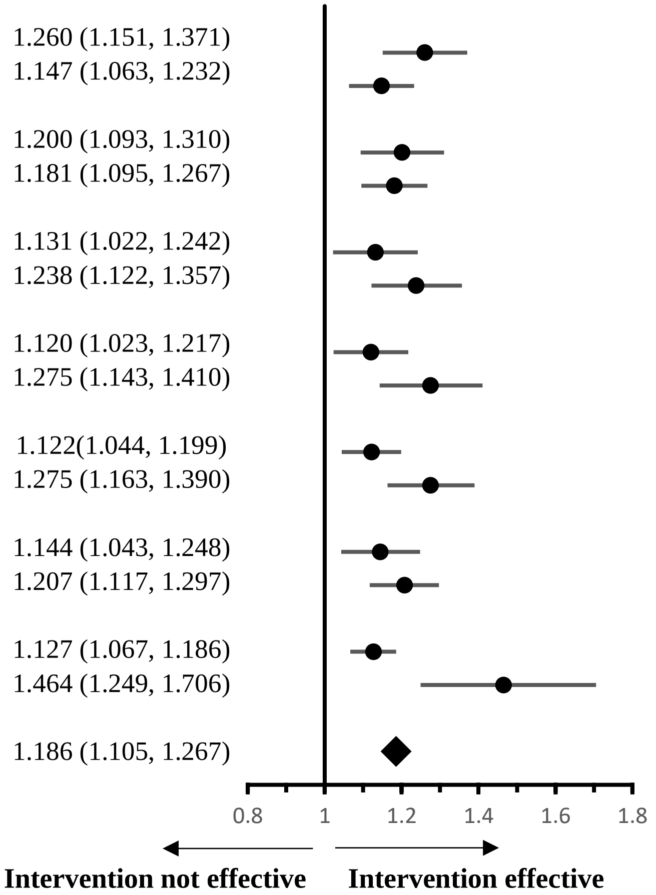

S1 Fig. Forest of subgroup analysis for primary outcome - HbA1c<7% at end of study

Note: Relative risk (95% CI) are derived from the odds ratio using the primary model: Logistic regression with GEE and with adjustment of baseline HbA1c. The p values are from the interaction term of subgroup variable and the intervention indicator.
