# Supplementary material for: Evaluation of an mHealth-enabled hierarchical diabetes management intervention in primary care in China (ROADMAP): A cluster randomized trial
Source: PLoS Med. 2021 Sep 21;18(9):e1003754. doi: 10.1371/journal.pmed.1003754 (PMC8454951; doi:10.1371/journal.pmed.1003754)
Supplement: S2 Fig — BP, blood pressure; FBG, fasting blood glucose; ROADMAP, Road to Hierarchical Diabetes Management at Primary Care Settings in China. (PDF) [file pmed.1003754.s003.pdf]

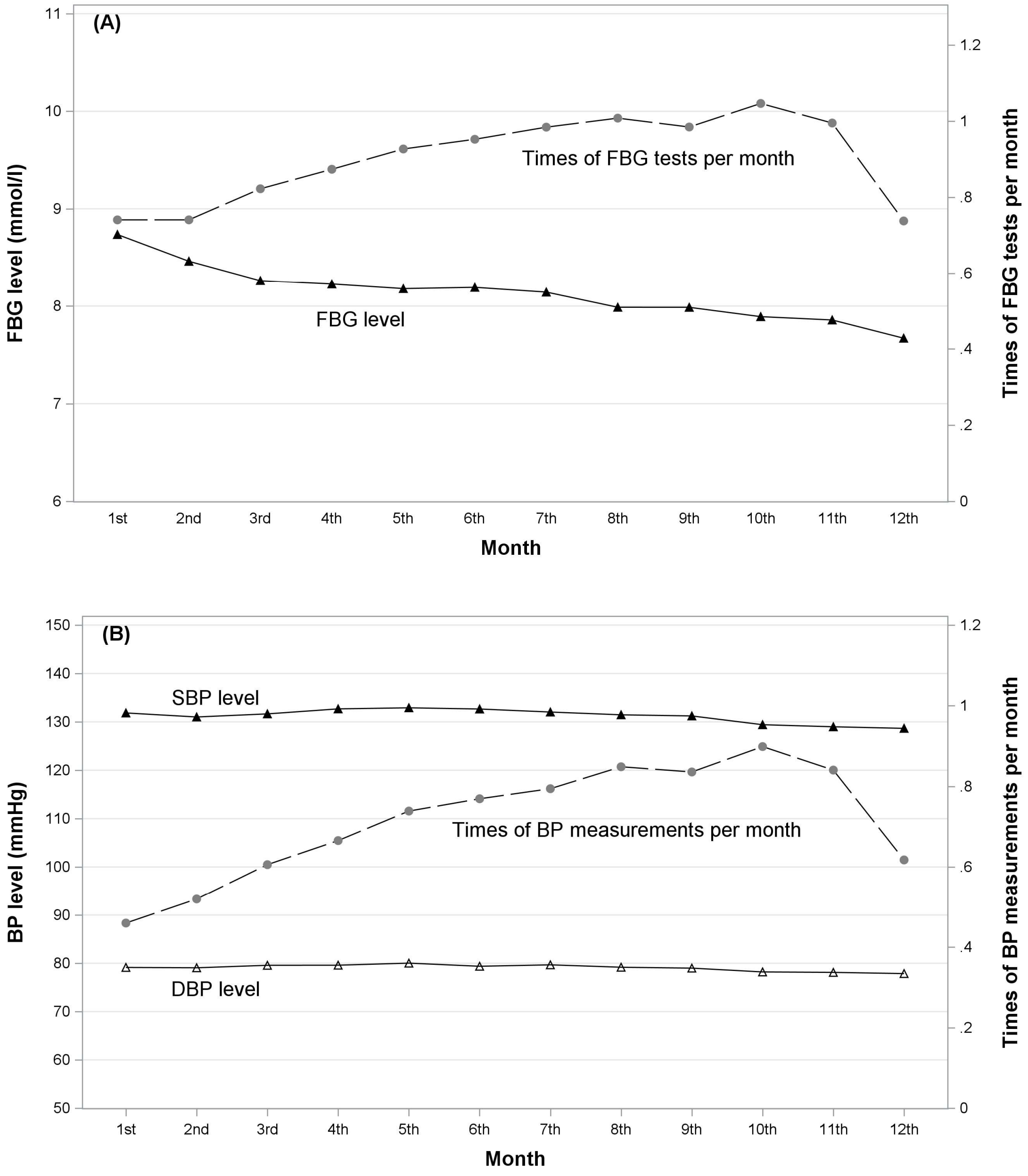

S2 Fig. The relationship between frequency of BP/FBG monitoring and BP/FBG control across 12 months in intervention group of ROADMAP study

Note: Data were collected from Graded ROADMAP App. BP: blood pressure; SBP: systolic blood pressure; DBP: diastolic blood pressure; FBG: fasting blood glucose tested by blood glucose meter connected to the Graded ROADMAP App and automatically uploaded through the App when conducted at primary care clinics.
