# Supplementary material for: Evaluation of an mHealth-enabled hierarchical diabetes management intervention in primary care in China (ROADMAP): A cluster randomized trial
Source: PLoS Med. 2021 Sep 21;18(9):e1003754. doi: 10.1371/journal.pmed.1003754 (PMC8454951; doi:10.1371/journal.pmed.1003754)
Supplement: S1 Table — (DOCX) [file pmed.1003754.s004.docx]

**S1 Table. Sensitivity analysis for estimated effect (binary outcomes) of intervention compared to control**

| **Outcomes** | **Covariates-adjusted model** *** | |  | **After multiple imputation** *†* | |
| --- | --- | --- | --- | --- | --- |
|  | **RR (95% CI)** | **RD (95% CI)** |  | **RR (95% CI)** | **RD (95% CI)** |
| **HbA1c level <7.0%** | 1.192  (1.112, 1.273) | 7.2  (4.2, 10.2) |  | 1.178  (1.103, 1.255) | 6.7  (3.9, 9.6) |
| **FBG < 7.0 mmol/L** | 1.069  (0.999, 1.139) | 2.8  (0.0, 5.7) |  | 1.069  (1.005, 1.134) | 2.8  (0.2, 5.5) |
| **BP < 140/80 mmHg** | 1.038  (0.980, 1.097) | 1.7  (-0.8, 4.2) |  | 1.021  (0.965, 1.078) | 0.9  (-1.5, 3.4) |
| **BP < 130/80 mmHg** | 1.041  (0.978, 1.105) | 1.8  (-1.0, 4.5) |  | 1.028  (0.968, 1.089) | 1.2  (-1.38, 3.85) |
| **LDL-c < 2.6 mmol/L** | 0.988  (0.919, 1.058) | -0.5  (-3.7, 2.7) |  | 0.992  (0.928, 1.056) | -0.4  (-3.3, 2.6) |
| **Composite ABC**  **control** *‡* | 1.219  (1.063, 1.395) | 2.0  (0.6, 3.5) |  | 1.196  (1.044, 1.367) | 1.8  (0.4, 3.3) |
| **Composite AB'C**  **control** *§* | 1.217  (1.038, 1.424) | 1.9  (0.3, 3.8) |  | 1.193  (1.021, 1.391) | 1.7  (0.20, 3.5) |

*Notes: FPG: fasting blood glucose. BP: blood pressure. SBP: systolic blood pressure. DBP: diastolic blood pressure. LDL-C: low-density lipoprotein cholesterol. RR: relative risk. RD: risk difference. CI: confidence interval.*

**: Logistic regression with GEE with further adjusted for age (<60,>=60), gender (male/female), economic developed level, locality (urban or rural) based on the primary model. RR are indirectly derived from OR using the formula: RR = OR ÷ [1 − p0 in control × (1-OR)].*

*†: Ten sets of imputed data were created and analysed using the primary model, then the estimates of the treatment effect (beta and its standard error) were combined to obtain the pooled common RR and RD along with their 95% CIs.*

*‡: Composite ABC control: defined as HbA1c level <7.0%, BP < 140/80 mmHg and LDL-C < 2.6 mmol/L.*

*§: Composite AB'C control: defined as HbA1c level <7.0%, BP < 130/80 mmHg and LDL-C < 2.6 mmol/L.*
