# Supplementary material for: Evaluation of an mHealth-enabled hierarchical diabetes management intervention in primary care in China (ROADMAP): A cluster randomized trial
Source: PLoS Med. 2021 Sep 21;18(9):e1003754. doi: 10.1371/journal.pmed.1003754 (PMC8454951; doi:10.1371/journal.pmed.1003754)
Supplement: S2 Table — (DOCX) [file pmed.1003754.s005.docx]

**S2 Table. Sensitivity analysis for estimated effect (continuous outcomes) of intervention compared to control**

|  | **Covariates-adjusted model** *** | | **After multiple imputation** *†* | |
| --- | --- | --- | --- | --- |
|  | **Mean Difference (95% CI)** | **P values** | **Mean Difference (95% CI)** | **P values** |
| **HbA1c level, %** | -0.30 ( -0.38, -0.21) | <0.001 | -0.27 ( -0.35, -0.19) | <0.001 |
| **FBG level, mmol/L** | -0.48 ( -0.62, -0.33) | <0.001 | -0.44 ( -0.58, -0.30) | <0.001 |
| **SBP, mmHg** | -0.95 ( -1.94, 0.03) | 0.058 | -0.84 ( -1.74, 0.07) | 0.070 |
| **DBP, mmHg** | -0.63 ( -1.17, -0.09) | 0.021 | -0.56 ( -1.05, -0.07) | 0.026 |
| **LDL-C level, mmol/L** | -0.01 ( -0.06, 0.05) | 0.828 | -0.00 ( -0.05, 0.04) | 0.855 |
| **Weight, kg** | -0.00 ( -0.19, 0.18) | 0.966 | 0.01 ( -0.17, 0.19) | 0.916 |

*Notes: FPG: fasting blood glucose. BP: blood pressure. SBP: systolic blood pressure. DBP: diastolic blood pressure. LDL-C: low-density lipoprotein cholesterol.*

**: Linear regression with GEE and further adjusted for age (<60,>=60), gender (male/female), economic developed level, locality (urban or rural).*

*†: Ten sets of imputed data were created and analysed using the primary model, then the estimates of the treatment effect (beta and its standard error) were combined to obtain the pooled common RR and RD along with their 95% CIs*
