# Supplementary material for: Evaluation of an mHealth-enabled hierarchical diabetes management intervention in primary care in China (ROADMAP): A cluster randomized trial
Source: PLoS Med. 2021 Sep 21;18(9):e1003754. doi: 10.1371/journal.pmed.1003754 (PMC8454951; doi:10.1371/journal.pmed.1003754)
Supplement: S3 Table — (DOCX) [file pmed.1003754.s006.docx]

**S3 Table. Comparison of primary and secondary binary outcomes between *Your Doctor* inactive and active users within intervention group**

|  | **Raw data, n (%)** | | | |  | **Propensity score model** *** | |
| --- | --- | --- | --- | --- | --- | --- | --- |
|  | **Inactive users** | | **Active users** | |  | **RR (95% CI)** | **P value** |
| **HbA1c < 7.0%** |  |  |  |  |  |  |  |
| Baseline | 4638 | (37.1%) | 180 | (34.4%) |  |  |  |
| EOS | 4921 | (43.8%) | 199 | (40.6%) |  | 1.117 (0.968, 1.267) | 0.126 |
| **FBG < 7.0 mmol/L** |  |  |  |  |  |  |  |
| Baseline | 4577 | (36.6%) | 172 | (32.8%) |  |  |  |
| EOS | 4982 | (44.4%) | 178 | (36.6%) |  | 0.913 (0.781, 1.051) | 0.213 |
| **BP < 140/80 mmHg** |  |  |  |  |  |  |  |
| Baseline | 5113 | (40.9%) | 248 | (47.3%) |  |  |  |
| EOS | 5070 | (45.1%) | 212 | (43.3%) |  | 0.911 (0.789, 1.038) | 0.170 |
| **LDL-C < 2.6 mmol/L** |  |  |  |  |  |  |  |
| Baseline | 5111 | (40.9%) | 240 | (45.9%) |  |  |  |
| EOS | 4987 | (44.8%) | 248 | (50.9%) |  | 1.224 (1.078, 1.366) | 0.003 |
| **Composite diabetes control** |  |  |  |  |  |  |  |
| Baseline | 948 | (7.6%) | 53 | (10.1%) |  |  |  |
| EOS | 1204 | (10.9%) | 54 | (11.1%) |  | 1.040 (0.739, 1.444) | 0.820 |

*Notes: EOS: end of study. FPG: fasting blood glucose. BP: blood pressure. LDL-C: low-density lipoprotein cholesterol. Composite diabetes control: defined as HbA1c level <7.0%, BP < 140/80 mmHg and LDL-C < 2.6 mmol/L. RR: relative risk. CI: confidence interval.*

**: Logistic regression with GEE, with adjustment of the baseline value of the analysed outcome, and with inverse propensity score weighted. The propensity score estimated from a simple logistic regression for probability of receiving the treatment the participant received, with the baseline covariates including age, sex, education, duration of diabetes, the baseline value of the outcome, diabetic complication, economic development level, and locality (urban or rural) as independent variables.*
