# Supplementary material for: Evaluation of an mHealth-enabled hierarchical diabetes management intervention in primary care in China (ROADMAP): A cluster randomized trial
Source: PLoS Med. 2021 Sep 21;18(9):e1003754. doi: 10.1371/journal.pmed.1003754 (PMC8454951; doi:10.1371/journal.pmed.1003754)
Supplement: S4 Table — EOS, end of study. (DOCX) [file pmed.1003754.s007.docx]

**S4 Table. Number of patients and episodes of hypoglycaemia within one month before end-of-study**

| **Category of hypoglycaemia** | **Control (n=5794)** | |  | **Intervention (n=11760)** | | **P value** * |
| --- | --- | --- | --- | --- | --- | --- |
|  | **Patients**  **n (%)** | **Episodes**  **n (per 100 patients)** |  | **Patients**  **n (%)** | **Episodes**  **n (per 100 patients)** |  |
| Symptomatic hypoglycaemia | 228 (3.9) | 347(6.0) |  | 457 (3.9) | 621 (5.3) | 0.821 |
| Asymptomatic hypoglycaemia | 167 (2.9) | 250 (4.3) |  | 310 (2.6) | 489 (4.2) | 0.615 |
| Probable symptomatic hypoglycaemia | 467 (8.1) | 1015 (17.5) |  | 979 (8.3) | 2093 (17.8) | 0.612 |
| Relative hypoglycaemia | 192 (3.3) | 314 (5.4) |  | 466 (4.0) | 813 (6.9) | 0.657 |
| Overall hypoglycaemia | 722 (12.5) | 1926 (33.2) |  | 1489 (12.7) | 4016 (34.1) | 0.825 |

**: P values are for comparison of the hypoglycemia episodes between two arms, which from Poisson regression with GEE and with adjustment of baseline count of each hypoglycemia.*
