# Supplementary material for: Evaluation of an mHealth-enabled hierarchical diabetes management intervention in primary care in China (ROADMAP): A cluster randomized trial
Source: PLoS Med. 2021 Sep 21;18(9):e1003754. doi: 10.1371/journal.pmed.1003754 (PMC8454951; doi:10.1371/journal.pmed.1003754)
Supplement: S5 Table — ROADMAP, Road to Hierarchical Diabetes Management at Primary Care Settings in China. (DOCX) [file pmed.1003754.s008.docx]

**S5 Table. Comparison of key diabetes management activities between two groups during one-year follow-up in ROADMAP study**

|  | **Control group (N=5794)** | **Intervention group (N=11760)** | **P value** |
| --- | --- | --- | --- |
| **Intervention activities through *Graded ROADMAP* App during follow-up (times/person/year) *** | | | |
| FBG test | - | 10.8 (7.6) | - |
| Post-prandial BG test | - | 7.9 (4.7) | - |
| BP measurement | - | 8.6 (4.7) | - |
| Patient referral applied | - | 0.25 (0.73) | - |
| Patient referral completed | - | 0.19 (0.65) | - |
| Active user of *Your Doctor* | - | 524 (4.0%) | - |
| **Self-reported times of examinations during follow-up (times/person/year), Mean (SD)** † | | | |
| Weight | 4.3 (13.6) | 5.8 (14.7) | <0.001 |
| BP | 16.2 (40.1) | 18.3 (37.4) | <0.001 |
| FBG | 18.2 (32.8) | 20.9 (32.1) | <0.001 |
| Post-prandial BG | 10.0 (23.0) | 14.5 (24.3) | <0.001 |
| HbA1c | 1.2 (1.4) | 1.4 (1.6) | <0.001 |
| Blood lipid | 1.1 (1.0) | 1.2 (1.1) | <0.001 |
| Foot: pulsation of the dorsal artery | 0.8 (1.4) | 1.1 (2.0) | <0.001 |
| Neurological examination | 0.4 (0.8) | 0.9 (1.7) | <0.001 |
| **Score of SDSCA, mean (SD)** ‡ | | | |
| Baseline | 27.7 (11.2) | 27.7 (11.4) | 0.983 |
| EOS | 29.0 (11.3) | 30.2 (11.2) | <0.001 |
| Change | 1.3 (13.5) | 2.5 (13.7) | 0.008 |
| Estimated mean difference (95% CI) | 1.2 (0.3, 2.0) | |  |
| **Medication §** |  |  |  |
| Proportion of participants receiving insulin therapy among all participants, n (%) | | | |
| Baseline | 1542 (23.7%) | 3206 (24.6%) | 0.168 |
| EOS | 1516 (26.2%) | 3192 (27.2%) | 0.171 |
| Averaged number of OADs used among participants with insulin-naïve therapy during follow-up, n, mean (SD) | | | |
| Baseline | 4060, 1.24 (0.77) | 8099, 1.25 (0.77) | 0.226 |
| EOS | 4060, 1.30 (0.82) | 8099, 1.34 (0.81) | 0.010 |

*Notes: Data are mean (SD) or n (%), unless otherwise specified. FBG: fasting blood glucose. BG: blood glucose. BP: blood pressure. SDSCA: Summary of Diabetes Self-Care Activities. EOS: end-of-study. OAD: oral antihyperglycemic drug*

**: Only including examinations and referrals conducted through Graded ROADMAP App.*

*†: The examinations could be* *tests and measurements conducted at home, primary care clinics and hospitals. P values are from Student t-test.*

*‡:* *Total score of SDSCA covering diet, exercise, blood sugar testing and foot care, not including smoking. P values for baseline and EOS scores are from Student t-test, while for score change or estimated mean difference, it is based on the primary model:* *linear regression with GEE accounting for clustering and baseline score.*

*§:* *The P values for proportion of participants receiving insulin therapy are from Chi-Square test, while those for OAD numbers are based on Student t- test.*
