# Supplementary material for: Evaluation of an mHealth-enabled hierarchical diabetes management intervention in primary care in China (ROADMAP): A cluster randomized trial
Source: PLoS Med. 2021 Sep 21;18(9):e1003754. doi: 10.1371/journal.pmed.1003754 (PMC8454951; doi:10.1371/journal.pmed.1003754)
Supplement: S6 Table — ROADMAP, Road to Hierarchical Diabetes Management at Primary Care Settings in China. (DOCX) [file pmed.1003754.s009.docx]

**S6 Table. Monthly performance of intervention implementation in ROADMAP study --- Results of 24 out of 144 counties in September 2018 as an example**

| **Province** | **County** | **# of Patients**  **recruited** | **Overall**  **rank** | **BG test** | | **BG control** | | **BP measurement** | | **BP control** | | **Referral** | |
| --- | --- | --- | --- | --- | --- | --- | --- | --- | --- | --- | --- | --- | --- |
|  |  |  |  | **Times/**  **person** | **Rank** | **Control rate of last FBG (%)** | **Rank** | **Times/**  **person** | **Rank** | **Control rate**  **of last BP (%)** | **Rank** | **Times/**  **person** | **Rank** |
| GX | LX | 88 | 3 | 2.11 | 11.5 | 44.32 | 27 | 1.06 | 23 | 51.14 | 13 | 0.13 | 5 |
| SD | LBKFQ | 90 | 4 | 4.32 | 1 | 54.44 | 12 | 1.88 | 1 | 66.67 | 2 | 0 | 80 |
| AH | LX | 93 | 9 | 2.01 | 22 | 55.91 | 10 | 1.23 | 5.5 | 43.01 | 26 | 0 | 80 |
| SD | QH | 99 | 13 | 1.91 | 38 | 41.41 | 35 | 0.96 | 47.5 | 42.42 | 28 | 0.01 | 23 |
| SC | WTQ | 88 | 14 | 2.05 | 16 | 42.05 | 33.5 | 0.98 | 39 | 56.82 | 7 | 0 | 80 |
| HN | TK | 97 | 20 | 1.73 | 59 | 45.36 | 23 | 1.18 | 12 | 27.84 | 78 | 0.01 | 23 |
| SC | RS | 90 | 23 | 2.13 | 9 | 53.33 | 15 | 0.6 | 102 | 33.33 | 55.5 | 0.01 | 23 |
| SX | LX | 88 | 25 | 1.94 | 35 | 26.14 | 90 | 0.88 | 66 | 45.45 | 20 | 0.25 | 3 |
| SX | BJCC | 91 | 26 | 2.09 | 13 | 37.36 | 43.5 | 1.04 | 25 | 34.07 | 54 | 0 | 80 |
| S_X | SY | 88 | 27 | 1.86 | 44 | 53.41 | 13.5 | 0.81 | 76.5 | 60.23 | 4.5 | 0 | 80 |
| LN | SYSJT | 95 | 29 | 2.07 | 14 | 38.95 | 39 | 0.95 | 49.5 | 37.89 | 45 | 0 | 80 |
| LN | TX | 89 | 30 | 2.01 | 22 | 38.2 | 40 | 0.93 | 53 | 39.33 | 38.5 | 0 | 80 |
| YN | ZTSLD | 89 | 34 | 1.81 | 50.5 | 37.08 | 45 | 1.09 | 17.5 | 37.08 | 48 | 0 | 80 |
| HB | HZ | 88 | 37 | 1.88 | 40.5 | 53.41 | 13.5 | 0.66 | 97.5 | 28.41 | 75.5 | 0.01 | 23 |
| LN | DGS | 90 | 41 | 2.03 | 17.5 | 44.44 | 25 | 1.03 | 26.5 | 17.78 | 103 | 0 | 80 |
| GS | ZYGZ | 87 | 41 | 1.97 | 30.5 | 39.08 | 38 | 0.91 | 59.5 | 37.93 | 44 | 0 | 80 |
| AH | TH | 88 | 47 | 1.84 | 46.5 | 26.14 | 90 | 0.97 | 43.5 | 52.27 | 10 | 0 | 80 |
| AH | PY | 88 | 52 | 1.99 | 26.5 | 25 | 93.5 | 0.99 | 36 | 38.64 | 42 | 0 | 80 |
| HB | EZ | 88 | 58 | 1.33 | 93 | 43.18 | 29 | 0.8 | 78 | 51.14 | 13 | 0 | 80 |
| HB | WHSDXH | 89 | 60 | 1.62 | 73 | 39.33 | 37 | 0.87 | 69.5 | 39.33 | 38.5 | 0 | 80 |
| GS | QY | 91 | 61 | 1.6 | 77 | 31.87 | 61 | 1.08 | 20 | 30.77 | 62 | 0 | 80 |
| YN | XW | 95 | 66 | 1.71 | 61 | 28.42 | 78 | 0.91 | 59.5 | 40 | 33 | 0 | 80 |
| YN | ZTDY | 90 | 67 | 1.62 | 73 | 22.22 | 101 | 1.22 | 7 | 34.44 | 52 | 0 | 80 |
| SD | JNYXYFS | 91 | 68 | 1.6 | 77 | 35.16 | 51 | 0.79 | 79 | 42.86 | 27 | 0 | 80 |
