# Supplementary material for: Evaluation of an mHealth-enabled hierarchical diabetes management intervention in primary care in China (ROADMAP): A cluster randomized trial
Source: PLoS Med. 2021 Sep 21;18(9):e1003754. doi: 10.1371/journal.pmed.1003754 (PMC8454951; doi:10.1371/journal.pmed.1003754)
Supplement: Attachment — Submitted filename: jia.pdf [file pmed.1003754.s010.pdf]

This paper reports a cluster trial of a complex intervention for diabetes management in China.

I was asked for a statistical report and I interpret that to include all aspects of the design and conduct of the study. The version I received did not have page numbers. My numbering has the abstract starting on page 2

Points of detail

Page 7 If the random allocation is in blocks within counties as the text suggests then I do not see the relevance of stratifying by economic development or urbanicity.

Page 9 I wonder whether a brief explanation of WeChat is needed for an international audience. It is not widely used outside China.

Page 10 I think the description of the sample size determination here is too brief. I know it is in the protocol but that demands the reader go off and find another document.

Page 11 The description of the main statistical model is rather confusing. First it is stated that it was planned to be a logistic regression and indeed that seems to be what the authors actually did. But we also read 'due to the planned non–convergence of the log–binomial regression'. So did the authors first try the log–binomial and then fall back on logistic when it failed to converge in which case 'planned' seems wrong? Or did they expect in advance that there might be problems when 'planned' perhaps means 'expected'?

Page 11 The imputation model is under–reported. What variables were included in the model and what was imputed? Many of the key variables were measured repeatedly during the study so could potentially have been used in the model along with baseline variables.

Page 11 If you run a Poisson regression for hypoglycaemia then it is the number of episodes which is being modelled, not the percentage.

Page 13 Although the attrition rates are small and roughly comparable it would be helpful to know about predictors of missingness and to have such variables included in the models.

Page 13 The $\pm$ notation is better avoided as ambiguous. In fact in Table 1 the authors use the much better mean (standard deviation) presentation.

Page 14 I might be easier to read if the percentages each followed the relevant condition

Page 15 For the record it is fine not to test for baseline differences.

Page 18 I think this is in fact Table 2, not 21. From this point onwards the numbering of all the tables seems to have gone awry including into the supplement. The figure numbering seems OK though.

Page 33 I would not have said that variables recorded by hospitals or primary care were self–reported.

Page 34 If the authors want to show that two variables co–vary why not plot them one against the other rather than giving two parallel plots.

Page 23 The two sentences starting 'Possible reasons for low fidelity' read as though the authors have information about the possible reasons but I do not see this reported in the results. Is this in fact supported by their data?

Point of more substance

Although the low uptake of the *Your Doctor* app is obviously disappointing I still think it is worth improving the analysis. The difference between app and no app is not cluster–randomised but is in design like a case–control study so can be analysed differently from the main analysis. The most pertinent contrast is one within clusters. Only the 178 clusters with both cases of app use and controls without app use are informative. For continuous outcomes the relevant approach would probably be a model with a random intercept for cluster and for binary outcomes conditional logistic regression. This would have the advantage of removing the influence of cluster level confounders like those mentioned on page 7 and also any unmeasured ones.

Summary

Quite a few suggestions for improvements in presentation and analysis.

Michael Dewey
