# Supplementary material for: Evaluation of an mHealth-enabled hierarchical diabetes management intervention in primary care in China (ROADMAP): A cluster randomized trial
Source: PLoS Med. 2021 Sep 21;18(9):e1003754. doi: 10.1371/journal.pmed.1003754 (PMC8454951; doi:10.1371/journal.pmed.1003754)
Supplement: Attachment — Submitted filename: Response to editor and reviewers.docx [file pmed.1003754.s011.docx]

**Requests from the editors:**

1. *Noting PLOS' data policy (https://journals.plos.org/plosmedicine/s/data-availability) please adapt your data statement to note that data will be made available from the data of publication.* *Please remove the sentence "... if the proposed use of data is adjudicated adequate to achieve the purpose.".*

**RESPONSE:** The sentence mentioned has been removed.

2. *Please adapt the title to better match journal style. We suggest "**Evaluation of an mHealth-enabled hierarchical diabetes management intervention in primary care in China (ROADMAP): A cluster-randomized trial".*

**RESPONSE:** Thanks, the suggested title has been adopted.

3. *Please adapt the abstract to a three-part structure. Please add a new final sentence to the "Methods and findings" subsection, which should begin "Study limitations include ..." or similar and quote 2-3 of the study's main limitations.*

**RESPONSE:** Yes, two limitations have been included at the end of “Methods and findings” subsection, which reads “Study limitations mainly include that it should be cautious when extrapolating the findings to general population with type 2 diabetes due to the participants in this study were recruited from registered patients in primary care; and apart from centralized test for HbA1c, other laboratory tests were conducted at local hospitals.”

4. *Please quote the study dates in the abstract. We suspect that "1717,754" contains an error - please correct.*

**RESPONSE:** Thanks for the reminding. The study dates have been quoted in the abstract as “from June 2017 to July 2018”. The “1717,754” is a mistake somehow. We have corrected it as “17,754”. Thanks again.

5. *Please quote aggregate demographic details for study participants in your abstract.*

**RESPONSE:** Some key demographic characteristics have been quoted in the abstract as “After one year follow-up, 17,754 participants (89.8%) completed the end-of-study assessment, with 45.1% of them from economically developed areas, 49.9% from urban areas, 60.5±8.4 years of age, 41.2% male, 6.0 years of median diabetes duration, and HbA1c level of 7.87%±1.92% (37.3% with HbA1c<7.0%) at baseline.”

6. *Please list secondary outcome measures in your abstract as appropriate.*

**RESPONSE:** The secondary outcomes have been summarized as “The secondary outcomes include the individual/combined control rates of blood glucose, blood pressure and LDL-C; changes in levels of HbA1c, blood pressure, LDL-C, fasting blood glucose and body weight; and episodes of hypoglycaemia.” In order not to expand the abstract too much, the cut-off values of the control rates were not included.

7. *Please make that "data were" in the abstract and any other instances.*

**RESPONSE:** Done. The manuscript was also checked throughout, no additional same instance was found.

8. *After the abstract we will need to ask you to add a new and accessible "Author summary" section in non-identical prose. You may find it helpful to consult one or two recent research papers in PLOS Medicine to get a sense of the preferred style.*

**RESPONSE:** The “Author summary” has been added after referring to several recently published papers.

9. *Please move the ethics information from the end of the text to the Methods section.*

**RESPONSE:** Done

10. *Throughout the text, please adapt reference call-outs to the following style: "... exist in China [13,14]." (noting the absence of spaces within the square brackets).*

**RESPONSE:** Done. We have accepted those changes in the tracked version of the manuscript for the purpose of easy reading.

11. *Please remove the information on funding, competing interests and data sharing from the end of the main text. In the event of publication, this information will appear in the article metadata, via entries in the submission form.*

**RESPONSE:** We have removed such information from the main text, left them in the submission form (article metadata). The corrected data sharing information following your suggestion about removing the sentence "... if the proposed use of data is adjudicated adequate to achieve the purpose" has been re-entered through the submission form.

PLEASE NOTE, as you did not ask to remove the “T[ransparency statement](http://www.bmj.com/content/347/bmj.f4796)”, we still keep it at the end of the main text. But if you think it is already covered in the cover letter or other materials, you can remove it from the main text by your own. Many thanks. It reads now “The Transparency statement: The corresponding author affirms that the manuscript is an honest, accurate, and transparent account of the study being reported; that no important aspects of the study have been omitted; and that any discrepancies from the study as planned have been explained.”

12. *Throughout the paper, please quote exact p values or "p<0.001".*

**RESPONSE:** Already checked the format of p values throughout and uniformed them as exact values (3 digitals) or “p<0.001” in Table 3, S1 Table and S4 Table.

13. *Please revisit the reference list to ensure that all citations match journal style. All italics should be converted into plain text; and 6 author names should be listed, where appropriate, rather than 3, followed by "et al.".*

**RESPONSE:** Revised.

14. *Please rename figure 1 "**Participant flowchart" or similar.*

**RESPONSE:** Renamed as “Fig 1. Participant flowchart”

15. *Please attach a completed CONSORT checklist, labelled "S1_CONSORT_Checklist" or similar and referred to by this label in your methods section. In the checklist, please refer to individual items by section (e.g., "Methods") and paragraph number rather than by line or page numbers, as the latter generally change in the event of publication.*

**RESPONSE:** The CONSORT Checklist has been completed, labelled as “S1 CONSORT Checklist”, and referred to at the beginning of Methods section.

**Comments from the reviewers:**

***** Reviewer #1:** (Michael Dewey)

*This paper reports a cluster trial of a complex intervention for diabetes management in China.*

*I was asked for a statistical report and I interpret that to include all aspects of the design and conduct of the study. The version I received did not have page numbers. My numbering has the abstract starting on page 2*

*Points of detail*

1. *Page 7 If the random allocation is in blocks within counties as the text suggests then I do not see the relevance of stratifying by economic development or urbanicity.*

**RESPONSE:** Agree. Stratifying by economic development or urbanicity at provincial level has no contribution to the randomization if the random allocation was already done within counties, the unit lower than province. Stratification here was not to do a stratified randomization, instead, we hope to find out the impact of economic development and urbanicity to the effect of mHealth intervention, which would be very meaningful to instruct the update of primary care in different situations. During the selection of study sites, we did our best to enrol similar number of counties among the four strata factorially formulated by economic developed/underdeveloped and urban/rural locality, with the purpose of having similar power to make conclusion for each stratum and achieving relative national representativeness. To make this purpose clearer, we add a phrase in the Randomization part of methods section. It reads “All participating counties were stratified by the economic development level of their affiliating provinces and urban/rural locality with the purpose of enrolling equal number of counties for each of the four strata.”

2. *Page 9 I wonder whether a* *brief explanation of WeChat is needed for an international audience. It is not widely used outside China.*

**RESPONSE:** Thanks for raising this. We have added a bracketed explanation after its first appearance in the Methods-Intervention sub-section: “…WeChat (the most popular app for communication, social media and mobile wallet in China)”

3. *Page 10 I think the description of the sample size determination here is too brief. I know it is in the protocol but that demands the reader go oﬀ and ﬁnd another document.*

**RESPONSE:** More clues has been added to the original introduction. Now, it reads “Briefly, 19,008 eligible patients from 864 communities (22 patients each) would be recruited at baseline, and 16,416 participants (14% drop rate) complete the EOS, which could provide an 89% power to detect a 5% absolute increase in the primary outcome for the intervention group. The sample size calculation assumed that 40% of participants would have well- controlled HbA1c (<7%) at EOS in the control group, with an intra- class correlation coefficient (ICC) of 0.15.”

4. *Page 11 The description of the main statistical model is rather confusing. First it is stated that it was planned to be a logistic regression and indeed that seems to be what the authors actually did. But we also read ‘due to the planned non–convergence of the log–binomial regression’. So did the authors ﬁrst try the log–binomial and then fall back on logistic when it failed to converge in which case ‘planned’ seems wrong? Or did they expect in advance that there might be problems when ‘planned’ perhaps means ‘expected’?*

**RESPONSE:** Sorry for the confusing description. We did try the log–binomial first and then fall back on logistic due to convergence problem, which has been pre-determined in our analysis plan (cited in the main text). We now have removed the “due to the planned non-convergence” and replaced it with a sentence “This was the pre-specified alternating model in case of non-convergence of the log-binomial regression” to explain why we used the logistic regression model."

5. *Page 11 The imputation model is under–reported. What variables were included in the model and what was imputed? Many of the key variables were measured repeatedly during the study so could potentially have been used in the model along with baseline variables.*

**RESPONSE:** Details regarding to “3) imputed analysis” has been made: “The multiple imputation (MI) technique using a fully conditional specification were adopted for the imputation. Ten sets of imputed data were created and analysed using the primary model and then pooled to estimate the treatment effect. When performing MI, the missing continuous values of HbA1c, FPG, blood pressure and LDL-C were imputed using linear regression, then dichotomized to get imputed binary outcomes. The variables used in the imputation model included patient characteristics including age, sex, education, economic development level, locality (urban/rural), duration of diabetes, key medical history and comorbidities; level of HbA1c, FPG, blood pressure and LDL-C at baseline; level of HbA1c, FPG, blood pressure and LDL-C at end of study; cluster indicator; and treatment (intervention/control).”

The published SAP said: “The imputation model will include the levels of HbA1c, FBG, BP, and LDL-C at 1 year and at baseline; a cluster indicator; a group indicator; and baseline variables including age, sex, education, duration of diabetes, medical history, comorbidities, concomitant medications, and resource use. Ten sets of imputed data will be created and analysed using the model 1 (described in “Primary Analyses”). HbA1c, FBG, BP, and LDL-C will first be imputed as continuous variables using linear regression and subsequently converted into binary variables. ……”

6. *Page 11 If you run a Poisson regression for hypoglycaemia then it is the number of episodes which is being modelled, not the percentage.*

**RESPONSE:** Thanks for the correction. We have modified the manuscript on page 11 accordingly: “The comparison of the number of hypoglycaemia episodes was tested using Poisson regression analysis with GEE and with adjustment of baseline episodes of each hypoglycaemia”.

As shown in S6 Table, both the numbers of episodes and percentage of patients were reported, and actually we did report the p value from the Poisson regression to test the episodes as showed by the footnote of this table: “P values are for comparison of the hypoglycaemia episodes between two arms, which from Poisson regression with GEE and with adjustment of baseline count of each hypoglycaemia”. We also slightly modified the header and column titles of S6 Table to make it clearer.

7. *Page 13 Although the attrition rates are small and roughly comparable it would be helpful to know about predictors of missingness and to have such variables included in the models.*

**RESPONSE:** Thanks. We compared all characteristics between participants with and without missing values for imputed variables including HbA1c and SBP (see tables below). We did not analyse the predictors to missingness of fasting blood glucose and LDL-C because they were based on the same blood sampling and the missingness status is the same as HbA1c. DBP had the same missing status as SBP and was not analysed for the predictors to missingness either. Standardized difference (SD) >0.1 was used to indicate if significant difference exists (Cohen J. Statistical Power Analysis for the Behavioural Sciences. 2nd ed: Lawrence Erlbaum Associates Publishers: Hillsdale, NJ 1988.). The results showed that nearly all the characteristics are comparable between patients with missing and non-missing HbA1c and SBP. Only smoking status (SD=0.154) for missingness of HbA1c and education level (SD=-0.102) for missingness of SBP were slightly imbalanced. However, although assuming missing at random, we still included all the covariates (including smoking and education level) adjusted in the covariates-adjusted model of outcome into the imputation model to improve the performance of the imputation. We also added a paragraph in manuscript to describe the details of the imputation and mention what variables be included in the imputation model: “When performing MI, the missing continuous values of HbA1c, FPG, blood pressure and LDL-C were imputed using linear regression, then dichotomized to get imputed binary outcomes. The variables used in the imputation model included patient characteristics including age, sex, education, economic development level, locality (urban/rural), duration of diabetes, key medical history and comorbidities; level of HbA1c, FPG, blood pressure and LDL-C at baseline; level of HbA1c, FPG, blood pressure and LDL-C at EOS; cluster indicator; and treatment (intervention/control).”

**Supporting table 1: Comparison between patients with and without missing HbA1C**

| **Characteristics** | **Non-missing group** | **Missing group** | **Standardized Difference** |
| --- | --- | --- | --- |
| **N** | 17514 | 2032 |  |
| **Age (years)** | 60.6 (8.3) | 60.1 (9.3) | -0.054 |
| **Male, n(%)** | 7103 (40.6%) | 957 (47.1%) | 0.024 |
| **BMI (kg/m^2)** | 25.7 (3.6) | 25.6 (3.6) | 0.056 |
| **Waist circumference (cm)** | 89.9 (9.6) | 90.3 (9.7) | -0.002 |
| **Han nationality, n(%)** | 16032 (91.5%) | 1802 (88.7%) | 0.048 |
| **Education level, n(%)** |  |  | -0.096 |
| **Current smokers, n(%)** | 3003 (17.1%) | 462 (22.7%) | 0.154 |
| **Health insurance covered, n(%)** | 17339 (99.0%) | 2004 (98.6%) | 0.025 |
| **Insurance reimbursement Rates, n(%)** |  |  | 0.025 |
| **Hypertension, n(%)** | 9084 (51.9%) | 1049 (51.6%) | -0.004 |
| **Dyslipidaemia , n(%)** | 5907 (33.7%) | 627 (30.9%) | -0.061 |
| **Diabetic nephropathy, n(%)** | 1240 (7.1%) | 150 (7.4%) | 0.012 |
| **Diabetic retinopathy, n(%)** | 2010 (11.5%) | 236 (11.6%) | 0.004 |
| **Peripheral neuropathy, n(%)** | 1307 (7.5%) | 147 (7.2%) | -0.009 |
| **Lower extremity, n(%)** | 819 (4.7%) | 95 (4.7%) | -0.000 |
| ^ ^ ^ Lower extremity artery disease, n(%) | 441 (2.5%) | 58 (2.9%) | 0.021 |
| ^ ^ ^ Peripheral vascular disease, n(%) | 234 (1.3%) | 28 (1.4%) | 0.004 |
| ^ ^ ^ Diabetic foot damage, n(%) | 313 (1.8%) | 28 (1.4%) | -0.033 |
| **Macrovascular, n(%)** | 3165 (18.1%) | 397 (19.5%) | 0.038 |
| ^ ^ ^ Carotid artery disease, n(%) | 868 (5.0%) | 99 (4.9%) | -0.004 |
| ^ ^ ^ Cardiac disease, n(%) | 892 (5.1%) | 107 (5.3%) | 0.008 |
| ^ ^ ^ ^ ^ ^ Coronary stenosis *, n(%) | 592 (3.4%) | 73 (3.6%) | 0.012 |
| ^ ^ ^ ^ ^ ^ Myocardial infarction, n(%) | 423 (2.4%) | 56 (2.8%) | 0.022 |
| ^ ^ ^ ^ ^ ^ Post coronary artery surgery **, n(%) | 323 (1.8%) | 37 (1.8%) | -0.002 |
| ^ ^ ^ Cerebral disease, n(%) | 1955 (11.2%) | 260 (12.8%) | 0.050 |
| ^ ^ ^ ^ ^ ^ Cerebral infarction, n(%) | 1544 (8.8%) | 204 (10.0%) | 0.042 |
| ^ ^ ^ ^ ^ ^ Cerebral haemorrhage, n(%) | 148 (0.8%) | 30 (1.5%) | 0.059 |
| ^ ^ ^ ^ ^ ^ Transient ischemic attack, n(%) | 481 (2.7%) | 55 (2.7%) | -0.002 |
| **Diabetic complications, n(%)** | 5698 (32.5%) | 664 (32.7%) | 0.003 |
| **All complications, n(%)** | 12216 (69.7%) | 1398 (68.8%) | -0.021 |
| **Duration of diabetes, years** | 7.4 (5.9) | 7.6 (6.1) | 0.037 |
| **Duration of hypertension, years** | 9.2 (7.4) | 9.3 (7.3) | 0.007 |
| **Duration of dyslipidaemia, years** | 6.0 (5.9) | 6.2 (5.8) | 0.024 |
| **Duration of diabetic nephropathy, years** | 5.7 (5.7) | 6.5 (7.2) | 0.121 |

**Supporting table 2: Comparison between patients with and without missing SBP**

| **Characteristics** | **All control group** | **All intervention group** | **Standardized Difference** |
| --- | --- | --- | --- |
| **N** | 17526 | 2020 |  |
| **Age (years)** | 60.6 (8.3) | 60.1 (9.2) | -0.055 |
| **Male, n(%)** | 7106 (40.5%) | 954 (47.2%) | 0.028 |
| **BMI (kg/m^2)** | 25.7 (3.6) | 25.6 (3.6) | 0.063 |
| **Waist circumference (cm)** | 89.9 (9.6) | 90.3 (9.7) | -0.003 |
| **Han nationality, n(%)** | 16046 (91.6%) | 1788 (88.5%) | 0.045 |
| **Education level, n(%)** |  |  | -0.102 |
| **^ ^ ^ Primary school or lower** | 9774 (55.8%) | 1096 (54.3%) |  |
| **^ ^ ^ Junior high school** | 5135 (29.3%) | 594 (29.4%) |  |
| **^ ^ ^ Senior high school** | 2000 (11.4%) | 259 (12.8%) |  |
| **^ ^ ^ Junior college** | 450 (2.6%) | 57 (2.8%) |  |
| **^ ^ ^ Bachelor's degree or higher** | 167 (1.0%) | 14 (0.7%) |  |
| **Current smoker, n(%)** | 3007 (17.2%) | 458 (22.7%) | 0.064 |
| **Health insurance covered, n(%)** | 17351 (99.0%) | 1992 (98.6%) | 0.028 |
| Hypertension, n(%) | 9089 (51.9%) | 1044 (51.7%) | -0.003 |
| Dyslipidaemia , n(%) | 5901 (33.7%) | 633 (31.4%) | -0.049 |
| Diabetic nephropathy, n(%) | 1240 (7.1%) | 150 (7.4%) | 0.014 |
| Diabetic retinopathy, n(%) | 2016 (11.5%) | 230 (11.4%) | -0.003 |
| Peripheral neuropathy, n(%) | 1309 (7.5%) | 145 (7.2%) | -0.011 |
| Lower extremity, n(%) | 819 (4.7%) | 95 (4.7%) | 0.001 |
| ^ ^ ^ Lower extremity artery disease, n(%) | 441 (2.5%) | 58 (2.9%) | 0.022 |
| ^ ^ ^ Peripheral vascular disease, n(%) | 234 (1.3%) | 28 (1.4%) | 0.004 |
| ^ ^ ^ Diabetic foot damage, n(%) | 313 (1.8%) | 28 (1.4%) | -0.032 |
| Macrovascular, n(%) | 3164 (18.1%) | 398 (19.7%) | 0.042 |
| ^ ^ ^ Carotid artery disease, n(%) | 868 (5.0%) | 99 (4.9%) | -0.002 |
| ^ ^ ^ Cardiac disease, n(%) | 890 (5.1%) | 109 (5.4%) | 0.014 |
| ^ ^ ^ ^ ^ ^ Coronary stenosis *, n(%) | 590 (3.4%) | 75 (3.7%) | 0.019 |
| ^ ^ ^ ^ ^ ^ Myocardial infarction, n(%) | 423 (2.4%) | 56 (2.8%) | 0.023 |
| ^ ^ ^ ^ ^ ^ Post coronary artery surgery **, n(%) | 322 (1.8%) | 38 (1.9%) | 0.003 |
| ^ ^ ^ Cerebral disease, n(%) | 1957 (11.2%) | 258 (12.8%) | 0.049 |
| ^ ^ ^ ^ ^ ^ Cerebral infarction, n(%) | 1546 (8.8%) | 202 (10.0%) | 0.041 |
| ^ ^ ^ ^ ^ ^ Cerebral haemorrhage, n(%) | 148 (0.8%) | 30 (1.5%) | 0.060 |
| ^ ^ ^ ^ ^ ^ Transient ischemic attack, n(%) | 481 (2.7%) | 55 (2.7%) | -0.001 |
| **Diabetic complications, n(%)** | 5701 (32.5%) | 661 (32.7%) | 0.004 |
| **All complications, n(%)** | 12221 (69.7%) | 1393 (69.0%) | -0.017 |

8. *Page 13 The ± notation is better avoided as ambiguous. In fact, in Table 1 the authors use the much better mean (standard deviation) presentation.*

**RESPONSE:** As suggested, we have replaced all ± notations throughout the manuscript with “mean (standard deviation)” presentation.

9. *Page 14 I might be easier to read if the percentages each followed the relevant condition*

**RESPONSE:** Thanks, the sentence has been re-written as “The self-reported prevalence for hypertension was 51.8%, dyslipidaemia 33.4%, macrovascular disease 18.2%, diabetic retinopathy 11.5%, diabetic nephropathy 7.1%, and diabetic peripheral neuropathy 7.4%.”

10. *Page 15 For the record it is ﬁne not to test for baseline diﬀerences.*

**RESPONSE:** Thanks.

11. *Page 18 I think this is in fact Table 2, not 21. From this point onwards the numbering of all the tables seems to have gone awry including into the supplement. The ﬁgure numbering seems OK though.*

**RESPONSE:** Yes, it might be due to a hidden format in the Word document or an issue when transforming from Doc to PDF format. We have re-written the table headers and checked the PDF version before the re-submission.

12. *Page 33 I would not have said that variables recorded by hospitals or primary care were self–reported.*

**RESPONSE:** Sorry for making you confused. It was self-reported times of examinations during follow-up. To avoid misunderstanding, we have refined the row title and footnote of S1 Table to emphasize that it is the “self-reported times of examinations” and “the examinations could be tests and measurements conducted at home, primary care clinics and hospitals”, respectively.

13. *Page 34 If the authors want to show that two variables co–vary why not plot them one against the other rather than giving two parallel plots.*

**RESPONSE:** Your advice is very helpful. We simplified the figure (S1 Fig) and made modification following your advice. To be specific, we removed one (% of patients testing FBG/BP>=1 time/month) of the three y-axis, only left “FBG/BP level” and “Times of FBG tests / BP measurements per month”, and using two lines to illustrate the co-vary of increasing number of FBG tests against the decrease of FBG level (part A), and the co-vary of increasing number of BP measurements against the decrease of BP levels (part B).

14. *Page 23 The two sentences starting ‘Possible reasons for low ﬁdelity’ read as though the authors have information about the possible reasons but I do not see this reported in the results. Is this in fact supported by their data?*

**RESPONSE:** The first sentence gives the causes for low fidelity according to other similar studies. The second one is the finding from our process evaluation, but it is difficult to be presented in the Results section. Now we further clarify the two statements as “Possible reasons **reported by other studies** for low fidelity in implementing a complex intervention include the need of longer learning time [45-47], competing workload [48], and not user friendly for elderly users [23]. Drawn from our **process evaluation**, however, fidelity issues were largely driven by low expectation of treat-to-target for blood glucose/ blood pressure by both patients and service providers, which resulted in negligence of alerts to abnormal blood glucose/ blood pressure measurements and referral indications by service providers and low patient compliance to instructions given by the providers.”

15. *Point of more substance*

*Although the low uptake of the Your Doctor app is obviously disappointing, I still think it is worth improving the analysis. The diﬀerence between app and no app is not cluster–randomised but is in design like a case–control study so can be analysed diﬀerently from the main analysis. The most pertinent contrast is one within clusters. Only the 178 clusters with both cases of app use and controls without app use are informative. For continuous outcomes the relevant approach would probably be a model with a random intercept for cluster and for binary outcomes conditional logistic regression. This would have the advantage of removing the inﬂuence of cluster level confounders like those mentioned on page 7 and also any unmeasured ones.*

**RESPONSE:** Hopefully we did not misunderstand you. We think a case–control study is to treat the end-point variable as the case or control, but in our study the app active user or non-active user are our explanatory factors rather than the end-point variable. Therefore, we think it will be not suitable to use “conditional logistic regression” from a case-control study for our binary outcomes. In this paper, we used propensity score method base on logistic regression model (for binary outcomes) with GEE or linear regression (for continuous outcomes) with GEE. It read in the manuscript: “Further comparison between active and inactive users of Your Doctor within the intervention group was conducted using an inverse propensity score weighted method based on the primary model.” The primary model here for binary outcomes referred to a logistic regression model with generalized estimating equations (GEE) accounting for clustering, and for continuous outcomes referred to a linear regression with GEE which is as similar as a random intercept for cluster.

So we didn’t perform the method you suggested. However, we also guess that you were suggesting compare the proportions of outcome in active user and non-active user only within the 178 clusters, using propensity score matching method. If so, we will try this method later as a sensitivity analysis. However, that will only leave 524 pairs from 178 clusters to contribute to the analysis and will lost a lot of information. To leverage all the data from the 576 intervention clusters, we still prefer presenting the results based on propensity score weighted method as defined by the established statistical analysis plan, which can as much as possibly utilize the information from all patients.

We are happy for a further communication if you have further comments on this. Many thanks.

16. *Summary*

*Quite a few suggestions for improvements in presentation and analysis.*

**RESPONSE:** All the suggestions are very helpful in improving the presentation and analysis. Many thanks for your thoughts and precious time.

***** Reviewer #2:**

*This is a very interesting paper. This outbreak of covid-19 is accelerating the progress of the development and promotion of the mHealth system, but there is no such large sample size of clinical research data in mHealth at present. This study can be seem to the international initiative and has great social effect, because the mHealth management caver in people with different living habits across regions and climates, and it also shows the ideal effect of blood glucose management. Therefore, it can be considered that the results of this study are acceptable to reflect the blood glucose management effect of mHealth system in real life. The grouping design, reasonable intervention measures and statistical results are highly reliable. Therefore, I suggest that it be published in the journal.*

**RESPONSE:** Thank you very much for affirming the importance of our research.

***** Reviewer #3: (**Mihiretu M. Kebede, PhD)

*This is a very well-written manuscript. I have few points for consideration.*

1. *Methods: the authors mentioned they have imputed data. Clarify the imputation methodology.*

**RESPONSE:** Similar question was also asked by Reviewer 2. The manuscript changes regarding to “imputed analysis” are: “The multiple imputation (MI) technique using a fully conditional specification were adopted for the imputation. Ten sets of imputed data were created and analysed using the primary model and then pooled to estimate the treatment effect. When performing MI, the missing continuous values of HbA1c, FPG, blood pressure and LDL-C were imputed using linear regression, then dichotomized to get imputed binary outcomes. The variables used in the imputation model included patient characteristics including age, sex, education, economic development level, locality (urban/rural), duration of diabetes, key medical history and comorbidities; level of HbA1c, FPG, blood pressure and LDL-C at baseline; level of HbA1c, FPG, blood pressure and LDL-C at end of study; cluster indicator; and treatment (intervention/control).”

Further information please refer to the response to Question 5 by reviewer 2 above.

2. *The intervention is not adequately described.*

**RESPONSE:** Against the CONSORT 2010 checklist of information reporting a randomised trial, we added some key information about (1) participants recruitment such as “Every six communities (i.e. clusters) were enrolled by county hospital investigators” and “In each community (cluster), an average of 22 participants were selected at random from a full list of T2D patients from local BPHS system.” (2) How and when the intervention components were actually administered, such as “The structural trainings at provincial and county level were delivered one by one by national and provincial working groups, respectively”; “County hospital investigators routinely oversaw the implementation of the contracted service following a designated operation manual”; “The performance assessment was conducted monthly by the national working group”; and “The Your Doctor app was provided to all the participants by their community doctors, but the app use was based on their willingness and capability in using a smartphone”. (3) A brief explanation of WeChat was also added as “…WeChat (the most popular app for communication, social media and mobile wallet in China)”

3. *Fig1 (trial profile): Both "Your doctor active" and "Your doctor inactive" show similar login values (>=4 logins per year) Majority of the participants are "Your doctor in active" users. Can you give a clear context why this happened? Highlight whether the active/inactive status makes a difference in the effects of the intervention.*

**RESPONSE:** Sorry for making such a mistake which might be caused by simple copy/paste. As stated in Methods, “Participants logged-in to this app for no less than four times throughout the intervention were defined as active *Your Doctor* users, while those with less than four logins were defined as inactive users.” The mistake in Figure 1 has been corrected accordingly. The effect comparison between active and inactive users was already shown in the Results part. It reads “The comparison of the binary outcomes between active and inactive users of *Your Doctor* among the intervention group is presented in Supplemental S5 Table. The primary multivariable regression model suggested no significant difference for blood glucose, blood pressure and composite diabetes control between the two sub-populations, apart from higher LDL-C control rate in the active users.”

4. *I was expecting the absolute HbA1c change to be one of the key outcomes. HbA1c change of 0.3 is clinically significant. This needs to be highlighted in the results section as one of the key results.*

**RESPONSE:** Following your suggestion, we have highlighted the HbA1c change of 0.3% in the results section and also mentioned in the first paragraph of Discussion section.

5. *Supplementary table shows diabetes self-care activities significantly gets improved. Please highlight that in the results section.*

**RESPONSE:** In the “Intervention Implementation” part of Results section, we added a sentence to highlight the improvement of total Score of Summary of Diabetes Self-Care Activities (SDSCA) after several sentences summarising the improvements in specific diabetes management activities. It reads “The improvement in these diabetes management activities in intervention group was also reflexed in the significant increase of total score of SDSCA by 1.2 (95% CI 0.3, 2.0) when compared to the control.”

In addition, when checking the statistics program, we found two places in S1 Table inappropriate and should be corrected. (1) Referring to the guidance by Kamradt et al. (Health and Quality of Life Outcomes (2014) 12:185), it is better to describe smoking status separately and not include it in the calculation of total score of SDSCA. (2) The *P* values should be from Student t- test for continuous variables, Chi-Square test for binary variables, and linear regression (primary model) for estimated mean difference of SDSCA score. The updates have been reflexed in the S1 Table. There is no significant difference between the updated and previous results.

6. *There are some typos across the manuscript. Please correct*

**RESPONSE:** Some typos have been corrected such as “Data was” as “Data were” and uniformed the English as US style.

7. *Abstract: Results section, please correct 1717, 754.*

**RESPONSE:** Many thanks. It has been corrected as 17,754

8. *Abbreviation ABC is not clear.*

**RESPONSE:** This has been clarified as HbA1c<7.0%, blood pressure<140/80 mmHg (in line with the Chinese diabetes guideline during the study) and LDL-C<2.6 mmol/L in both Abstract and Outcomes part of Methods sections. In the Methods section and S3 Table for sensitivity analysis, an alternative AB’C with 130/80 mmHg as BP targets was described to facilitate comparability with other studies.

9. *Change Table 21 to Table 1*

**RESPONSE:** Similar mistake also happened with Table 32. They should be Table 2 and Table 3. These errors might be due to a hidden field format in the Word document or an issue when transforming from Doc to PDF format. We have re-written the table headers and checked the PDF version before the re-submission.

***
