# Supplementary material for: Evaluation of an mHealth-enabled hierarchical diabetes management intervention in primary care in China (ROADMAP): A cluster randomized trial
Source: PLoS Med. 2021 Sep 21;18(9):e1003754. doi: 10.1371/journal.pmed.1003754 (PMC8454951; doi:10.1371/journal.pmed.1003754)
Supplement: Attachment — Submitted filename: Response to reviewers_PMEDICINE-D-21-00273R2 20210731-Clean.docx [file pmed.1003754.s012.docx]

**Comments from Reviewers:**

1. *Please adapt the information on seeking access to study data in the data statement, as "... following the instruction of NODE" may not be clear to some readers: would "Researchers can apply for access to anonymized study data and associated documents through NODE" be suitable wording?*

**RESPONSE:** Following your advice, we have replaced the sentence “Researchers could request the anonymized data and related documents following the instruction of NODE” in the article metadata with “Researchers can apply for access to anonymized study data and associated documents through NODE”.

2. *Please adapt the short title to "Cluster-randomized trial of diabetes management in China" or similar.*

**RESPONSE:** Many thanks. We have replaced the original short title “Diabetes management in China of a cluster-randomized controlled trial” with “Cluster-randomized trial of diabetes management in China” in the article metadata.

3. *In the abstract, we suggest adapting "... data were primarily analyzed" to "... data were analyzed".*

**RESPONSE:** We have removed the word “primarily” from the sentence of "... data were primarily analysed" in the abstract in the article metadata and the manuscript.

4. *Where you discuss the primary findings in the abstract, please adapt the phrasing to "...* *the intervention led to an absolute improvement in the HbA1c control rate of 7% (95% CI ...) and* *a relative improvement of 18.6% ... " or similar.*

**RESPONSE:** We have replaced the following sentence in the abstract

“Compared with usual care, the intervention improved HbA1c control rate by 7.0% (95% confidence interval [CI] 4.0% to 10.0%) absolutely and 18.6% (relative risk [RR] 1.186, 95% CI 1.105 to 1.267) relatively, and increased the composite ABC control (HbA1c<7.0%, blood pressure<140/80 mmHg and LDL-cholesterol<2.6 mmol/L) by 21.8% (RR 1.218, 95% CI 1.062 to 1.395)”

with

“Compared with usual care, the intervention led to an absolute improvement in the HbA1c control rate of 7.0% (95% confidence interval [CI] 4.0% to 10.0%) and a relative improvement of 18.6% (relative risk [RR] 1.186, 95% CI 1.105 to 1.267), and an absolute improvement in the composite ABC control (HbA1c<7.0%, blood pressure<140/80 mmHg and LDL-cholesterol<2.6 mmol/L) rate of 1.9% (95% CI 0.5 to 3.5) and a relative improvement of 21.8% (RR 1.218, 95% CI 1.062 to 1.395)”

in both the article metadata and manuscript.

5. *In the final sentence of the "Methods and findings" subsection of your abstract, please amend the text to "* *... and caution should be exercised when extrapolating ...".*

**RESPONSE:** Many thanks. We have replaced the expression of “it should be cautious when…” with “caution should be exercised when…” in both the article metadata and manuscript.

6. *In the Author summary, we suggest amending the first point to "**In many countries, health outcomes are impacted by the consequences of poor glycemic control among people living with diabetes owing to the low quality of primary care." or similar.*

**RESPONSE:** Many thanks. We have amended the first point of the Author summary in the manuscript according to your advice.

7. *Later on in this section, please make that "**To our knowledge, this study is the largest in testing ..." or similar.*

**RESPONSE:** Many thanks. We have amended the first point under “What do these findings mean?” in Author summary by replacing “This study is the largest in testing…” with “To our knowledge, this study is the largest in testing ...”.

8. *Please restructure the Results section so that the presentation of primary and secondary endpoint findings comes before the "Intervention implementation" subsection.*

**RESPONSE:** Following your advice and considering that “Hypoglycaemia and Weight Gain” and “Subgroup Analysis” are closely relevant to the primary/secondary outcomes, we have moved the “Intervention implementation” part to the end of “Results” section. Accordingly, we have renumbered the supplementary tables and figures in the manuscript and the supplementary files.

9. *Where you present the primary findings, at the start of p.17 of the PDF, you mention "... an absolute reduction of 7.0% (95% CI 4.0 - 10%)." Should this be an "absolute increase"?*

**RESPONSE:** Yes, it is. We have corrected it. Many thanks.

10. *Immediately before table 2 you quote an "absolute increase of 2.0%" for composite ABC control, whereas the table appears to quote this as 1.9%: please check that these are consistent.*

**RESPONSE:** After careful checking, we confirmed that it should be 1.9%, not 2.0%. We have corrected it.

11. *In the first paragraph of the Discussion, please quote the trial's primary endpoint findings before the discussion of the intervention components such as "Your Doctor".*

**RESPONSE:**

Your suggestion make sense. We have re-written the first paragraph of the Discussion as below:

“To our knowledge, ROADMAP is the largest randomized controlled trial in testing the effectiveness and safety of an mHealth-based diabetes management with a diverse coverage of primary care settings. After one-year follow-up, we found that the intervention lowered the HbA1c level by 0.3% when compared with usual care, which led to an absolute improvement in the HbA1c control rate of 7.0% (95% CI 4.0% to 10.0%) and a relative improvement of 18.6% (RR 1.186, 95% CI 1.105 to 1.267). We also observed an absolute improvement in the intervention group in the composite cardiometabolic ABC control rate of 1.9% (95% CI 0.5 to 3.5) and a relative improvement of 21.8% (RR 1.218, 95% CI 1.062 to 1.395). The increased control of FBG and blood pressure, and the reduced levels of FBG and DBP favored the intervention as well. No increasing episodes of hypoglycemia and weight gain were found to be associated with the intervention. The findings indicate that integrated mHealth solution which facilitates blood glucose monitoring and performance evaluation could be effective and safe in improving diabetes control in primary care settings.”

12. *In the final paragraph of the Discussion, please amend the first sentence to "In conclusion, the primary care system in China, as in other LMIC, is struggling ...".*

**RESPONSE:** Yes, we have amended the sentence accordingly. Many thanks.

13. *Throughout the text, please remove spaces from the reference call-outs (e.g., "... innovative technologies [13,15,16].").*

**RESPONSE:** Done.

14. *Please remove the information on competing interests from references 3, 5 and any other relevant references.*

**RESPONSE:** The information on competing interests has been removed from references 3, 5 and 13.

**Comments from Reviewers:**

*** Reviewer #1:

The authors have addressed my points

Michael Dewey

***
